# Trauma exposure and factors associated with ICD-11 PTSD and complex PTSD in the Lithuanian general population

**DOI:** 10.1177/00207640211057720

**Published:** 2021-11-18

**Authors:** Monika Kvedaraite, Odeta Gelezelyte, Agniete Kairyte, Neil P Roberts, Evaldas Kazlauskas

**Affiliations:** 1 Center for Psychotraumatology, Institute of Psychology, Vilnius University, Lithuania; 2 National Centre for Mental Health (NCMH), Division of Psychological Medicine and Clinical Neurosciences, Cardiff University School of Medicine, Cardiff, UK; 3 Directorate of Psychology and Psychological Therapies, Cardiff & Vale University Health Board, Cardiff, UK

**Keywords:** ICD-11, trauma, PTSD, CPTSD, trauma disclosure

## Abstract

**Background:**

After the inclusion of a novel diagnosis of Complex Posttraumatic Stress Disorder (CPTSD) in the 11th edition of the International Classification of Diseases (ICD-11), there is a growing need for research focused on not only studying the underlying risk factors of this disorder but also differentiating the risk factors of Posttraumatic Stress Disorder (PTSD) and CPTSD to understand better the factors leading to CPTSD onset and symptom maintenance.

**Aims:**

This study aimed to explore the prevalence of traumatic experiences, trauma-related disorders and risk factors associated with ICD-11 PTSD and CPTSD in a population-based Lithuanian sample using the International Trauma Questionnaire (ITQ).

**Methods:**

The study sample included 885 participants (age *M*[*SD*] = 37.96 [14.67], 63.4% female). The Life Events Checklist was used to measure trauma exposure, PTSD and CPTSD symptoms were measured by the Lithuanian ITQ version. The Disclosure of Trauma Questionnaire (DTQ) was used to measure the urge or reluctance to talk about trauma.

**Results:**

The prevalence of at least one traumatic experience in the study sample was 81.4%. The prevalence of PTSD and CPTSD among the general population in Lithuania was 5.8% and 1.8%, respectively. Accumulative lifetime trauma exposure, sexual assault and assault with a weapon were significant predictors for both PTSD and CPTSD. Participants from the CPTSD group reported greater reluctance to disclose trauma and stronger emotional reactions than no diagnosis and PTSD groups. Results also indicate that the Lithuanian ITQ version is a valid measure for screening PTSD and CPTSD in the general population.

**Conclusion:**

Previous history of trauma and interpersonal trauma were associated with posttraumatic stress disorders but did not differentiate between PTSD and CPTSD in our study. However, social trauma-related factors, such as trauma disclosure, were associated with stronger CPTSD symptoms.

## Introduction

The inclusion of a new diagnosis of Complex Posttraumatic Stress Disorder (CPTSD) in the 11th edition of the International Classification of Diseases (ICD-11) (World Health Organization, 2018) calls for comprehensive research and validation of possible assessment tools and exploration of risk factors to better understand the new diagnosis. The most widely used measure for ICD-11 CPTSD is the self-report International Trauma Questionnaire (ITQ; Cloitre et al., 2018). The ITQ measures the three core posttraumatic stress disorder (PTSD) symptoms – (1) re-experience, (2) avoidance and (3) current sense of threat – and the functional impairment caused by these symptoms. In addition, the ITQ measures the three core symptoms of disturbances in self-organisation (DSO) – (1) affect dysregulation, (2) negative self-concept and (3) disturbances in relationships and DSO symptom-related functional impairment (Cloitre et al., 2018).

The ITQ has been validated in various samples, including the general population, clinical and veteran samples (Cloitre et al., 2019; Hyland et al., 2019; Karatzias et al., 2019) and is the best currently available measure of ICD-11 CPTSD. There is also a growing number of studies using the translated versions of the ITQ (Folke et al., 2019; Ho et al., 2019; Maercker et al., 2018; Mordeno et al., 2019), enabling CPTSD studies in various cultures. However, although most studies have supported the ITQ validity, there are differences in symptom structure in different samples (Redican et al., 2021). Hence, translation to other languages and exploring the validity of the ITQ across cultures is highly relevant, considering the ICD-11 will be implemented in healthcare from 2022. Furthermore, to our knowledge, there is also a lack of studies showing the measurement invariance of the ITQ across age or gender.

The current study was conducted in Lithuania, a European Union country that, despite a high prevalence of trauma exposure based on empirical findings (Kazlauskas & Zelviene, 2016), is struggling to provide adequate treatment for PTSD in healthcare (Kazlauskas, 2017). In the context of the ICD-11 updates of PTSD and CPTSD definitions, the only published Lithuanian study exploring trauma exposure and risk factors of PTSD and CPTSD in an adult population is in the clinical sample (Kazlauskas et al., 2018). However, that study did not use the final version of the ITQ, but one including additional items for testing the structural validity (Kazlauskas et al., 2018).

An important research line following the inclusion of a novel diagnosis of CPTSD in ICD-11 is studying the underlying risk factors for CPTSD, which is essential for both prevention and intervention of CPTSD. Given the nature of CPTSD, which includes all three core PTSD symptoms and additional DSO symptoms (World Health Organization, 2018), CPTSD could be more disabling and have a more chronic course than PTSD. Furthermore, differentiating the risk factors of PTSD and CPTSD could lead to a better understanding of CPTSD onset and symptom maintenance and would help in providing appropriate treatment. Previous research on PTSD shows that peri- and post-trauma risk factors have the most substantial impact on the onset of PTSD (Brewin et al., 2000; Ozer et al., 2003). Among the most researched risk factors are trauma and social-related factors such as trauma type, previous trauma, trauma severity, disclosure of trauma and social support.

A growing number of studies explores the link between these risk factors and CPTSD, showing that childhood or sexual trauma is one of the strongest predictors of CPTSD, as well as cumulative trauma and lack of social support (Cloitre et al., 2019; Hansford & Jobson, 2021; Kazlauskas et al., 2018; Knefel et al., 2019; Krammer et al., 2016; Simon et al., 2019). However, the majority of these studies have been conducted in the US or UK, and the results indicate that there could be substantial differences in trauma-related risk factors across different populations and settings (Palic et al., 2016). Furthermore, to our knowledge, there is a lack of studies exploring the relationship between disclosure of trauma and CPTSD, even though studies conducted in the context of PTSD shows that disclosure of trauma could have a robust therapeutic effect (Gradus, 2017). Traumatic experiences more commonly associated with CPTSD, such as interpersonal violence, could lead to more negative adverse emotional reactions such as shame or guilt (Cunningham, 2020), which are shown to inhibit trauma disclosure. Therefore CPTSD related trauma experiences could lead to a poorer negative self-concept, more adverse psychopathological reactions and poorer treatment outcomes (Bedard-Gilligan et al., 2012; MacGinley et al., 2019). Hence, it is important to have a greater understanding of the relationship between trauma disclosure and CPTSD.

This study aimed to validate the Lithuanian version of the ITQ and assess whether this instrument is suitable for screening for ICD-11 PTSD and CPTSD reactions regardless of age or gender. Furthermore, we aimed to explore the prevalence of traumatic experiences and trauma-related disorders in Lithuania and risk factors associated with PTSD and CPTSD. We explored gender and age effects as well as trauma-related risk factors of PTSD/CPTSD, such as the recency of trauma, trauma type, cumulative trauma and the disclosure of trauma as these factors are shown to be related to stronger psychopathology following traumatic experiences (Brewin et al., 2000).

## Methods

### Participants and procedure

This study was a part of a larger trauma and mental health study conducted by the Center for Psychotraumatology, Vilnius University. It was approved by the Institutional Psychological Research Ethics Committee. Data collection took place between July 2015 and December 2017 and was collected by 63 trained interviewers (53 psychologists and 10 trained psychology students). Inclusion criteria for this study were: (1) ⩾18 years old; (2) understanding of the Lithuanian language. Participants were recruited at various locations in Lithuania (e.g. home, work, community centres settings) throughout the country, including urban and rural areas. Overall, 1,146 people were invited to participate in this study – 78.9% of them agreed, of which 77.2% fully completed the survey. All participants provided informed written consent before completing the questionnaires.

In total, 885 participants were included in the current study, of which 561 (63.4%) were female, mean age was 37.96 (*SD* = 14.67), ranging from 18 to 85 years. The majority of study participants were from an urban area (*n* = 712, 80.5%). More detailed sociodemographic characteristics of the sample are presented in Table 1. The majority of participants (*n* = 720, 81.4%) were exposed to at least one traumatic event during their lifetime, and 51 (5.8%) met the probable diagnostic criteria for PTSD and 16 (1.8%) – for CPTSD.

**Table 1.** Sociodemographic characteristics of the sample (*N* = 885).

Variable	Total sample (*n =* 885)	No diagnosis (*n* = 818)	PTSD (*n* = 51)	CPTSD (*n* = 16)	Significance statistics
	*n* (%)	*n* (%)	*n* (%)	*n* (%)	
Gender					
Male	324 (36.6)	311 (38.0)	10 (19.6)	3 (18.7)	χ^2^(2) = 9.25**
Female	561 (63.4)	507 (62.0)	41 (80.4)	13 (81.3)	
Age					
Mean (*SD*)	37.96 (14.67)	38.06 (14.70)	38.04 (14.49)	32.50 (13.59)	*F*(2) = 1.13
Range	18–85	18–85	19–76	21–63	
Relationship status^a^					
In a committed relationship	589 (66.6)	556 (68.0)	27 (54.0)	6 (37.5)	χ^2^(2) = 10.93***
Not in a committed relationship	289 (32.7)	256 (31.3)	23 (46.0)	10 (62.5)	
Children^a^					
Yes	494 (55.8)	453 (55.4)	32 (62.7)	9 (56.3)	χ^2^(2) = 1.04
No	390 (44.1)	364 (44.5)	19 (37.3)	7 (43.8)	
Residence^a^					
Urban	712 (80.5)	657 (80.3)	43 (84.3)	12 (75.0)	χ^2^(2) = 0.76
Rural	169 (19.1)	157 (19.2)	8 (15.7)	4 (25.0)	
Education					
University degree	396 (44.7)	364 (44.5)	27 (52.9)	5 (31.3)	χ^2^(4) = 4.95
Professional or technical school	318 (35.9)	297 (36.4)	16 (31.4)	5 (31.3)	
High school or lower	171 (19.3)	157 (19.2)	8 (15.7)	6 (37.5)	
Employment^a^					
Employed	673 (76.0)	623 (76.2)	40 (78.4)	10 (62.5)	χ^2^(2) = 1.89
Unemployed	205 (23.2)	188 (23.0)	11 (21.6)	6 (37.5)	
Income in Euros^a^					
Average or higher	491 (55.5)	456 (55.7)	25 (52.1)	5 (35.7)	χ^2^(2) = 1.26
Lower than average	372 (42.0)	340 (41.6)	23 (47.9)	9 (64.3)	

*Note.* χ^2^ = Chi-square statistics; *F* = variation between sample means.

a Results calculated with missing data (<3%).

** *p* < .01. ***p < .001.

### Measures

The revised version of the Life Events Checklist (LEC-R) was used to measure trauma exposure during the lifetime (Weathers et al., 2013). LEC-R is comprised of 16 potentially traumatic events (e.g. natural disaster, assault) with two additional items added to the standard version measuring: (1) physical abuse in childhood and (2) sexual abuse in childhood. Participants had to indicate whether the traumatic event ‘Happened to me’, ‘Witnessed it’, ‘Learned about it’, ‘Not sure’ and ‘Doesn’t apply to me’. Exposure to trauma was considered if the participants either experienced the event themselves or witnessed it. The sum of all traumatic experiences was used to estimate cumulative trauma exposure. The Lithuanian version of LEC-R was used in several studies previously (Kvedaraite et al., 2020; Truskauskaite-Kuneviciene et al., 2020).

The International Trauma Questionnaire (ITQ) was used to measure PTSD and CPTSD based on ICD-11 criteria (Cloitre et al., 2018). The ITQ is comprised of two parts – PTSD and DSO, constituting of the three symptom clusters each, with two items per cluster. PTSD clusters as defined in the ICD-11 are re-experiencing (Re), avoidance (Av) and sense of threat (Th); and three DSO symptom clusters are affective dysregulation (AD), negative self-concept (NSC) and disturbances in relationships (DR). Functional impairment regarding social life, occupational or any other important part of life was measured twice – for both PTSD and CPTSD symptoms. All ITQ items were rated on a five-point scale from 0 (=Not at all) to 4 (=Extremely) in association with the index traumatic event. The endorsement of a symptom cluster or functional impairment is defined as a score of ⩾2. According to the diagnostic algorithm (Cloitre et al., 2018) of the ITQ, the diagnosis of PTSD requires the endorsement of at least one of two symptoms from each PTSD cluster and the endorsement of functional impairment related to these symptoms. A diagnosis of CPTSD requires all criteria for PTSD and the endorsement of at least one of two symptoms from each of DSO clusters, plus the endorsement of functional impairment related to these symptoms. The internal reliability of the ITQ scale in a trauma-exposed group was found to be good – McDonald’s omega for the total ITQ score was 0.86, for PTSD and DSO symptom scores McDonald’s omega was 0.85 and 0.77, respectively.

The Disclosure of Trauma Questionnaire (DTQ-12) was used to measure avoidance of trauma disclosure (Müller & Maercker, 2006). The DTQ-12 comprise 12 items forming three subscales: (1) Reluctance to talk, (2) Urge to talk and (3) Emotional reactions, with four items per subscale. Participants were asked to respond according to how they felt about each item in relation to the experienced index traumatic event and were asked to rate each item on a six-point scale ranging from 0 (=I agree not at all) to 5 (=I agree completely). Total scores for the three subscales were calculated by adding all item’s scores included in the subscales. Higher urge to talk subscale scores indicates greater disclosure of traumatic experience. In contrast, higher Emotional reactions and Reluctance to talk subscales scores indicate greater difficulty to talk about the traumatic experiences. Previous studies using the Lithuanian version of DTQ demonstrated good internal reliability of this measure (Kvedaraite et al., 2020). McDonald’s omega of the DTQ scale in this study was 0.78 and varied from 0.77 to 0.82 for the subscales.

### Data analysis

The data analyses were conducted using IBM SPSS version 25.0 and the Mplus version 8.2. To test the factor structure of the ITQ, we conducted Confirmatory Factor Analysis (CFA). In this analysis, we tested four factor models tested in previous studies (Kazlauskas et al., 2018, 2020). The CFA models were estimated using the Robust Maximum Likelihood (MLR) estimator. The model fit in CFA analysis was evaluated by using the Comparative Fit Index (CFI), the Tucker–Lewis Index (TLI) and the Root Mean Square Error of Approximation (RMSEA), following the goodness of fit recommendation provided by Kline (2011). Namely, CFI/TLI values higher than 0.90 indicated an acceptable fit, and values higher than 0.95 represented a good fit; RMSEA values below 0.08 indicated an acceptable fit, and values <0.05 suggested a good fit. The measurement invariance test was used to check whether the ITQ scale can be used for both genders (female vs. male) and across different age groups, such as emerging adults (18–29 years old) and older (>29 years). Model comparisons were conducted by examining the changes in fit indices, where ΔCFI ⩾0.010 supplemented by ΔRMSEA ⩾0.015 were indicative of the significant difference between models (Chen, 2007). To test the reliability of the measurements, we computed McDonald’s omega reliability coefficients (McDonald, 1978).

## Results

### Validity of the ITQ

The psychometric properties of the ITQ in the general population sample were good. In line with the previous studies (Cloitre et al., 2018, 2021; Ho et al., 2019; Owczarek et al., 2020), the CFA results confirmed a correlated second-order two-factor model to be the best fit, where a second-order PTSD factor accounts for the covariation between the Re, Av and Th factors and a second-order DSO factor accounts for the covariation between the AD, NSC and DR factors (χ^2^(47) = 162.62, *p* < .001; CFI/TLI = 0.970/0.958; RMSEA [90% CI] = 0.058 [0.049–0.068]; SRMR = 0.041). All factor loadings in the CFA model were significant at *p* < .001 and ranged from 0.36 to 0.96. The standardised factor loading of the first-order AD factor on the second-order DSO factor was 1.18. This could be explained by multicollinearity, but it is not indicative of model misspecification (Deegan, 1978). The standardised factor correlation between PTSD and DSO was 0.58 (*p* < .001).

The scalar age measurement invariance and the partial scalar gender measurement invariance were established by allowing for the intercepts of one ITQ scale item (DR2 ‘Disturbed Relationships – Feeling Close to Other’) to vary across gender groups (see Table 2).

**Table 2.** Results of the ITQ measurement invariance tests by gender and age groups in the trauma-exposed sample (*n* = 720).

	Model fit indices			Model comparisons	
	χ^2^ (*df*)	CFI	RMSEA [90% CI]	ΔCFI	ΔRMSEA
Gender					
Configural	166.57 (78)	0.967	0.056 [0.044–0.068]		
Metric	178.98 (84)	0.965	0.056 [0.045–0.067]	0.002	0.000
Scalar	218.19 (90)	0.953	0.063 [0.052–0.074]	0.014	0.007
Partial scalar	166.95 (79)	0.968	0.056 [0.044–0.067]	0.001	0.000
Age					
Configural	143.86 (78)	0.976	0.048 [0.036–0.061]		
Metric	144.79 (84)	0.978	0.045 [0.032–0.057]	0.003	0.002
Scalar	160.43 (90)	0.974	0.047 [0.035–0.058]	0.001	0.002

*Note.* ITQ = International Trauma Questionnaire; χ^2^ = Chi-square goodness of fit statistics; *df* = degrees of freedom; CFI = Comparative Fit Index; RMSEA [90% CI] = root mean square error of approximation with 90% confidence intervals.

### Trauma exposure in the general population

The exposure to various traumatic experiences in the total sample and by gender and age are presented in Table 3. The majority of the study sample (81.4%) reported exposure to at least one traumatic event in their lifetime. Participants reported 3.41 (*SD* = 2.17) lifetime types of trauma exposure on average, ranging from zero to 18 events. The most common traumatic experiences in our sample were transportation accidents (42.6%), physical assault (40.0%) and sudden accidental death of a loved one (28.7%).

**Table 3.** Lifetime traumatic experiences and age and gender effects (*N* = 885).

	Total sample	Gender			Age		
		Male	Female	χ^2^(1)	18–29 years	>29 years	χ^2^(1)
	*n* (%)	*n* (%)	*n* (%)		*n* (%)	*n* (%)	
1. Natural disaster	55 (6.2)	26 (8.0)	29 (5.2)	2.87	32 (9.5)	23 (4.2)	9.93**
2. Fire or explosion	192 (21.7)	77 (23.8)	115 (20.5)	1.29	75 (22.2)	117 (24.4)	0.08
3. Transportation accident	377 (42.6)	163 (50.3)	214 (38.1)	12.43***	152 (45.0)	225 (41.1)	1.26
4. Serious other accident	205 (23.2)	98 (30.2)	107 (19.1)	14.41***	84 (24.9)	121 (22.1)	0.88
5. Exposure to toxic substance	53 (6.0)	29 (9.0)	24 (4.3)	7.97**	21 (6.5)	32 (5.9)	0.05
6. Childhood physical abuse	205 (23.2)	85 (26.2)	120 (21.4)	2.71	85 (25.1)	120 (21.9)	1.21
7. Physical assault	354 (40.0)	184 (56.8)	170 (30.3)	60.04***	153 (45.3)	201 (36.7)	6.32*
8. Assault with a weapon	75 (8.5)	43 (13.3)	32 (5.7)	15.16***	37 (10.9)	38 (6.9)	4.31*
9. Childhood sexual abuse	21 (2.4)	4 (1.2)	17 (3.0)	2.86	5 (1.5)	16 (2.9)	1.89
10. Sexual assault	29 (3.3)	2 (0.6)	27 (4.8)	11.41***	7 (2.1)	22 (4.0)	2.51
11. Other unwanted sexual experience	66 (7.5)	11 (3.4)	55 (9.8)	12.22***	25 (7.4)	41 (7.5)	0.00
12. Combat or exposure to a war-zone	15 (1.7)	12 (3.7)	3 (0.5)	12.38***	5 (1.5)	10 (1.8)	1.15
13. Captivity	10 (1.1)	7 (2.2)	3 (0.5)	4.86*	4 (1.2)	6 (1.1)	0.01
14. Life-threatening illness or injury	198 (22.4)	71 (21.9)	127 (22.6)	0.06	72 (21.3)	126 (23.0)	0.36
15. Severe human suffering	258 (29.2)	72 (22.2)	186 (33.2)	11.89***	93 (27.5)	165 (30.2)	0.71
16. Sudden violent death	60 (6.8)	22 (6.8)	38 (6.8)	0.00	18 (5.3)	42 (7.7)	1.83
17. Sudden accidental death	259 (28.7)	86 (26.5)	168 (29.9)	1.16	91 (26.9)	163 (29.8)	0.84
18. Serious injury, harm or death caused to someone else	29 (3.3)	19 (5.9)	10 (1.8)	10.80***	11 (3.3)	18 (3.3)	0.0

*Note.* χ^2^ = Chi-square statistics.

* *p* < .05. **p < .01. ***p < .001.

We found significant differences in the prevalence of traumatic events between genders, with males participants reporting more lifetime trauma exposure (*M* = 3.12, *SD* = 2.51) than females (*M* = 2.26, *SD* = 2.26) (*t*(883) = −3.32, *p* = .001). Male participants reported higher exposure to transportation accidents, serious other accidents (at work, home or during recreational activities), exposure to toxic substances, physical assault, assault with a weapon, combat or exposure to a warzone, captivity and serious injury, harm or death caused to someone else (see Table 3). Sexual assault, other unwanted or uncomfortable sexual experiences and severe human suffering were more frequently reported by females than males (see Table 3). We also found significant differences in the prevalence of traumatic events between the two analysed age groups. The participants in the emerging adulthood group (18–29 years old) reported higher exposure to natural disasters, physical assault and assault with a weapon than older participants.

### Prevalence and trauma-related predictors of PTSD and CPTSD in the general population

In the general population sample, 51 (5.8%) participants met the diagnostic criteria for PTSD and 16 (1.8%) – for CPTSD. We found no significant age effect but there was significant gender effect on both ICD-11 PTSD and CPTSD diagnosis with 80.4% (*n* = 41) female as compared to 19.6% (*n* = 10) male participants in PTSD group and 81.3% (*n* = 13) female as compared to 18.7% (*n* = 3) male participants in CPTSD group (see Table 1).

Descriptive statistics for all PTSD and CPTSD symptoms are presented in Table 4. PTSD symptoms in the PTSD and CPTSD groups were higher in comparison to the no diagnosis group. Furthermore, PTSD and DSO symptoms were greater in the CPTSD group than in the PTSD group (see Table 4).

**Table 4.** Means and standard deviations of PTSD and CPTSD symptoms in the trauma-exposed group (*n* = 720).

Variable	No diagnosis (*n* = 653)	PTSD (*n* = 51)	CPTSD (*n* = 16)	Significance statistics
	*M* (*SD*)	*M* (*SD*)	*M* (*SD*)	*F*(2)
Total ITQ symptom score	8.47 (6.54)^b,c^	21.45 (5.15)^a,c^	30.69 (4.95)^a,b^	182.31***
PTSD symptoms	3.82 (3.96)^b,c^	14.35 (3.21)^a^	16.19 (3.90)^a^	240.28***
Re-experiencing	1.00 (1.52)^b,c^	4.22 (1.55)^a,c^	5.38 (1.96)^a,b^	161.73***
Avoidance	1.43 (1.95)^b,c^	4.88 (1.56)^a^	5.75 (1.77)^a^	110.93***
Sense of threat	1.38 (1.72)^b,c^	5.26 (1.64)^a^	5.06 (1.61)^a^	152.31***
DSO symptoms	4.65 (4.02)^b,c^	7.10 (3.28)^a,c^	14.50 (2.78)^a,b^	55.80***
Affective dysregulation	1.99 (1.52)^b,c^	3.00 (1.49)^a,c^	4.06 (1.29)^a,b^	23.79***
Negative self-concept	1.02 (1.69)^c^	1.51 (1.33)^c^	5.63 (1.26)^a,b^	61.34***
Disturbed relationships	1.64 (1.85)^b,c^	2.59 (1.94)^a,c^	4.81 (1.60)^a,b^	28.15***
Trauma disclosure				
Reluctance to talk	6.03 (4.24)^b,c^	7.82 (4.48)^a,c^	11.40 (3.75)^a,b^	15.27***
Urge to talk	7.48 (4.53)^b^	9.98 (4.24)^a^	9.13 (3.54)	8.13***
Emotional reactions	6.27 (4.29)^b,c^	11.30 (4.39)^a,c^	14.69 (3.00)^a,b^	59.72***

*Note.* ITQ = International Trauma Questionnaire; *F* = variance between sample means.

a,b,c Significant differences at *p* < .05 (^a^No diagnosis, ^b^PTSD, ^c^CPTSD groups).

*** *p* < .001.

Cumulative lifetime trauma exposure was a significant predictor for both PTSD (OR = 1.16) and CPTSD (OR = 1.24) diagnostic status in contrast to no diagnosis, while exposure to a recent traumatic event significantly predicted PTSD (OR = 2.45), but not CPTSD (see Table 5). Assault with a weapon (OR = 2.77), sexual assault (OR = 4.22), sudden violent death of a loved one (OR = 3.01) and sudden accidental death of a loved one (OR = 1.98) were significant predictors of higher risk of PTSD (see Table 5). Assault with a weapon (OR = 4.58), sexual assault (OR = 7.30), other unwanted or uncomfortable sexual experiences (OR = 6.40), life-threatening illness or injury (OR = 2.80) and severe human suffering (OR = 8.47) were significant predictors of higher risk of CPTSD (see Table 5).

**Table 5.** Univariate logistic regression analysis for traumatic experiences as predictors of ICD-11 PTSD and CPTSD in trauma-exposed participants (*n* = 720).

Traumatic experiences	PTSD versus no diagnosis (*n* = 704)		CPTSD versus no diagnosis (*n* = 669)	
	OR [95% CI]	*p*	OR [95% CI]	*p*
1. Natural disaster	0.24 [0.03–1.86]	.160	0.76 [0.10–5.83]	.787
2. Fire or explosion	1.34 [0.74–2.44]	.337	1.28 [0.44–3.74]	.650
3. Transportation accident	0.95 [0.55–1.62]	.842	0.90 [0.33–2.42]	.832
4. Serious other accident	0.73 [0.37–1.44]	.361	1.49 [0.53–4.15]	.451
5. Exposure to toxic substance	1.82 [0.75–4.46]	.188	0.88 [0.11–6.80]	.902
6. Childhood physical abuse	1.74 [0.96–3.12]	.066	2.69. [0.99–7.27]	.051
7. Physical assault	1.31 [0.78–2.24]	.307	0.62 [0.22–1.74]	.367
8. Assault with a weapon	2.77 [1.35–5.68]	.005	4.58 [1.54–13.62]	.006
9. Childhood sexual abuse	1.44 [0.33–6.39]	.631	2.35 [0.29–18.78]	.420
10. Sexual assault	4.22 [1.61–11.03]	.003	7.30 [1.93–27.67]	.003
11. Other unwanted sexual experience	0.91 [0.32–2.61]	.857	6.40 [2.24–18.24]	.001
12. Combat or exposure to a war-zone	0.91 [0.12–7.08]	.931	–	–
13. Captivity	–	–	–	–
14. Life-threatening illness or injury	1.53 [0.84–2.78]	.168	2.80 [1.13–7.57]	.043
15. Severe human suffering	1.74 [0.98–3.08]	.059	8.47 [2.39–30.04]	.001
16. Sudden violent death	3.01 [1.42–6.37]	.004	0.82 [0.11–6.35]	.851
17. Sudden accidental death	1.98 [1.12–3.51]	.019	0.44 [0.12–1.56]	.212
18. Serious injury, harm or death caused to someone else	1.03 [0.24–4.46]	.973	3.59 [0.78–16.65]	.103
Sum of all traumatic events	1.16 [1.04–1.29]	.010	1.24 [1.06–1.45]	.008
Exposure to a recent traumatic event (<12-months)	2.45 [1.30–4.61]	.005	1.87 [0.59–5.96]	.292

*Note.* OR = odds ratio; CI = confidence interval.

We also found significant differences in the disclosure of traumatic events between no diagnosis, PTSD and CPTSD groups (see Table 4). Participants from the PTSD group reported stronger reluctance to talk about traumatic events and stronger emotional reactions while disclosing than the no diagnosis group. In comparison, participants from the CPTSD group reported stronger reluctance and stronger emotional reactions than those with no diagnosis or PTSD. The results also show that participants from the PTSD group indicated a stronger urge to talk about the traumatic event than the no diagnosis group.

## Discussion

This was one of the first studies which analysed trauma exposure prevalence and ICD-11 PTSD and CPTSD prevalence in the Lithuanian general sample. In total, 885 people agreed to participate in our study, and the response rate of 77% is similar to other studies of stress-related disorders conducted in the general Lithuanian population (Zelviene et al., 2020). We found a high prevalence of trauma exposure in the sample in line with previous studies (Kazlauskas & Zelviene, 2016). In the total sample, PTSD prevalence was 5.8% and CPTSD −1.8%, and is broadly comparable to findings in other countries (Ben-Ezra et al., 2018) in non-clinical samples, although population-based studies undertaken in different countries have produced variable prevalence rates (Cloitre et al., 2019; Maercker et al., 2018). Furthermore, we identified various PTSD and CPTSD predictors, particularly trauma and trauma disclosure related predictors relevant to future studies and clinical practice.

This study adds to the growing body of research on the ICD-11 PTSD and CPTSD, showing that the ITQ is an acceptable tool to measure posttraumatic stress reactions. In line with previous studies (Cloitre et al., 2018, 2021; Ho et al., 2019; Owczarek et al., 2020) and consistent with ICD-11 diagnostic criteria for PTSD and CPTSD (World Health Organization, 2018), we found that the two-factor (PTSD and DSO) second-order model demonstrated the best fit for the Lithuanian version of ITQ. Furthermore, using configural, metric and scalar measurement invariance testing, the study also showed that the ITQ could be used to screen for PTSD and CPTSD symptoms among different adult age groups. The gender invariance measurement indicated issues in the use of the ITQ among female and male populations, particularly regarding the item measuring DSO ‘close relationships with others’ symptoms. However, the partial scalar invariance showed that the ITQ could be used to measure PTSD and CPTSD in both genders. Consistent with a large body of previous research, our results also showed that female gender is associated with a higher risk of developing posttraumatic stress disorders (Ditlevsen & Elklit, 2012; Pineles et al., 2017).

This study aimed to assess the role of trauma-related factors, such as the type of trauma and the disclosure of traumatic events, on the onset of PTSD and CPTSD. In line with previous studies (Karatzias et al., 2017), various types of interpersonal trauma were related to PTSD and CPTSD symptoms, mainly sexual assault and assault with a weapon significantly increased the risk of both PTSD and CPTSD reactions in the current study. However, the results of this study did not find a significant association between childhood abuse (physical or sexual) and CPTSD, even though these findings have been reported previously (Cloitre et al., 2009; Kazlauskas et al., 2018; Knefel et al., 2019; Krammer et al., 2016). The current study replicates previous findings that PTSD is more strongly associated with recent trauma exposure than CPTSD (Karatzias et al., 2019). However, our study findings show that the cumulative effect of trauma exposure increased the risk of not only CPTSD but PTSD as well. These results are highly important in the clinical setting, suggesting that the type of trauma or the cumulative effect of trauma may be regarded only as a guiding factor but should not be used to determine the possible diagnosis or differentiate between PTSD or CPTSD symptoms.

A novel aspect of this study is the finding that avoidance of trauma disclosure is strongly associated with CPTSD symptoms, as disclosure of trauma has been sparsely studied in the context of ICD-11 CPTSD diagnosis. Previous studies have shown that avoidance of trauma disclosure can lead to stronger PTSD reactions (Bolton et al., 2003; Maercker & Horn, 2013), but the current study shows that it is a more substantial factor when talking about CPTSD reactions. Our results indicate that a higher risk of CPTSD is more strongly related to adverse reactions to trauma-related stimuli, such as strong reluctance to talk about the negative experiences and having strong emotional reactions when prompted to disclose them. Strong reluctance to disclose may be particularly related to the experience of negative social emotions such as shame and humiliation, which are often associated with interpersonal traumas such as sexual assault and sexual abuse, and could have undermining consequences as it could lead to reluctance to seek professional help (Kazlauskas, 2017) and more adverse psychopathology.

Compared to other studies that showed that disclosure of trauma is related to lower PTSD symptoms and could be used as an effective therapeutic method (Jeffreys et al., 2010), the current study showed that the urge to talk about trauma-related content is more strongly related to higher PTSD symptoms. A possible explanation for these results could be that disclosure of trauma when met with adverse social reactions from others could lead to even stronger PTSD reactions (Pielmaier & Maercker, 2011; Ullman & Filipas, 2001). Therefore, when exploring the effect of trauma disclosure on PTSD or CPTSD, it is important also to include the experience of perceived social support and social acknowledgement from others.

## Limitations

The current study provided important insights into assessing trauma-related disorders and trauma-related risk factors in the general population; however, it has several limitations that need to be considered when interpreting the findings. Firstly, the study is cross-sectional, limiting causal inferences and making the identified associations more challenging to interpret and susceptible to biases. Also, as this was not a clinical sample, PTSD and CPTSD groups were relatively small, so the estimation of predictors was limited to small statistical power. Therefore, the results of this study should be interpreted carefully. Moreover, even though our sample size was sufficient for a trustworthy data analysis, it was not a representative population-based study, meaning that the generalisation of our results for all population of Lithuania should be made with caution. Furthermore, we used self-report measures to assess the risk of PTSD and CPTSD. While the ITQ used in the current study is one of the most used measures for ICD-11 posttraumatic stress disorders, diagnostic clinical interviews, such as the International Trauma Interview (ITI), could provide more accurate diagnostic decisions (Bondjers et al., 2019) in future studies.

## Conclusions

All in all, the study provides insight into the role of trauma-related factors on PTSD and CPTSD in the general population. Our findings suggest that previous trauma and interpersonal trauma are important risk factors associated with posttraumatic stress disorders but may not differentiate between PTSD and CPTSD diagnosis, especially in non-clinical samples. However, this study highlights that CPTSD symptoms are related to adverse trauma disclosure, such as more substantial reluctance to disclose trauma history and having stronger emotional reactions, which could lead to the development of CPTSD and may be associated with reluctance to seek mental health services.

## References

[bibr1-00207640211057720] Bedard-Gilligan M. Jaeger J. Echiverri-Cohen A. Zoellner L. A. (2012). Individual differences in trauma disclosure. Journal of Behavior Therapy and Experimental Psychiatry, 43(2), 716–723. 10.1016/j.jbtep.2011.10.005 22080869 PMC3262897

[bibr2-00207640211057720] Ben-Ezra M. Karatzias T. Hyland P. Brewin C. R. Cloitre M. Bisson J. I. Roberts N. P. Lueger-Schuster B. Shevlin M. (2018). Posttraumatic stress disorder (PTSD) and complex PTSD (CPTSD) as per ICD-11 proposals: A population study in Israel. Depression and Anxiety, 35(3), 264–274. 10.1002/da.22723 29451956

[bibr3-00207640211057720] Bolton E. E. Glenn D. M. Orsillo S. Roemer L. Litz B. T. (2003). The relationship between self-disclosure and symptoms of posttraumatic stress disorder in peacekeepers deployed to Somalia. Journal of Traumatic Stress, 16(3), 203–210. 10.1023/A:1023754820991 12816331

[bibr4-00207640211057720] Bondjers K. Hyland P. Roberts N. P. Bisson J. I. Willebrand M. Arnberg F. K. (2019). Validation of a clinician-administered diagnostic measure of ICD-11 PTSD and complex PTSD: The international trauma interview in a Swedish sample. European Journal of Psychotraumatology, 10(1), 1665617. 10.1080/20008198.2019.1665617 31632616 PMC6781232

[bibr5-00207640211057720] Brewin C. R. Andrews B. Valentine J. D. (2000). Meta-analysis of risk factors for posttraumatic stress disorder in trauma-exposed adults. Journal of Consulting and Clinical Psychology, 68(5), 748–766. 10.1037/0022-006X.68.5.748 11068961

[bibr6-00207640211057720] Chen F. F. (2007). Sensitivity of goodness of fit indexes to lack of measurement invariance. Structural Equation Modeling: A Multidisciplinary Journal, 14(3), 464–504. 10.1080/10705510701301834

[bibr7-00207640211057720] Cloitre M. Hyland P. Bisson J. I. Brewin C. R. Roberts N. P. Karatzias T. Shevlin M. (2019). ICD-11 posttraumatic stress disorder and complex posttraumatic stress disorder in the United States: A population-based study. Journal of Traumatic Stress, 32(6), 833–842. 10.1002/jts.22454 31800131

[bibr8-00207640211057720] Cloitre M. Hyland P. Prins A. Shevlin M. (2021). The international trauma questionnaire (ITQ) measures reliable and clinically significant treatment-related change in PTSD and complex PTSD. European Journal of Psychotraumatology, 12(1), 1930961. 10.1080/20008198.2021.1930961 34211640 PMC8221157

[bibr9-00207640211057720] Cloitre M. Shevlin M. Brewin C. R. Bisson J. I. Roberts N. P. Maercker A. Karatzias T. Hyland P. (2018). The international trauma questionnaire: Development of a self-report measure of ICD-11 PTSD and complex PTSD. Acta Psychiatrica Scandinavica, 138(6), 536–546. 10.1111/acps.12956 30178492

[bibr10-00207640211057720] Cloitre M. Stolbach B. C. Herman J. L. van der Kolk B. Pynoos R. Wang J. Petkova E. (2009). A developmental approach to complex PTSD: Childhood and adult cumulative trauma as predictors of symptom complexity. Journal of Traumatic Stress, 22(5), 399–408. 10.1002/jts.20444 19795402

[bibr11-00207640211057720] Cunningham K. C. (2020). Shame and guilt in PTSD. In M. T. Tull & N. A. Kimbrel (Eds.), Emotion in posttraumatic stress disorder: Etiology, assessment, neurobiology, and treatment (pp. 145–171). Elsevier Academic Press.

[bibr12-00207640211057720] Deegan J. (1978). On the occurrence of standardized regression coefficients greater than one. Educational and Psychological Measurement, 38(4), 873–888. 10.1177/001316447803800404

[bibr13-00207640211057720] Ditlevsen D. N. Elklit A. (2012). Gender, trauma type, and PTSD prevalence: A re-analysis of 18 Nordic convenience samples. Annals of General Psychiatry, 11(1), 26. 10.1186/1744-859X-11-26 23107002 PMC3494556

[bibr14-00207640211057720] Folke S. Nielsen A. B. S. Andersen S. B. Karatzias T. Karstoft K. I. (2019). ICD-11 PTSD and complex PTSD in treatment-seeking Danish veterans: A latent profile analysis. European Journal of Psychotraumatology, 10(1), 1686806. 10.1080/20008198.2019.1686806 31762954 PMC6853202

[bibr15-00207640211057720] Gradus J. L. (2017). Prevalence and prognosis of stress disorders: A review of the epidemiologic literature. Clinical Epidemiology, 9, 251–260. 10.2147/CLEP.S106250 28496365 PMC5422316

[bibr16-00207640211057720] Hansford M. Jobson L. (2021). Social support and self-construal as moderators of lifetime trauma exposure on posttraumatic stress disorder symptoms. Traumatology, 27(2), 205–214. 10.1037/trm0000281

[bibr17-00207640211057720] Ho G. W. K. Karatzias T. Cloitre M. Chan A. C. Y. Bressington D. Chien W. T. Hyland P. Shevlin M. (2019). Translation and validation of the Chinese ICD-11 international trauma questionnaire (ITQ) for the assessment of posttraumatic stress disorder (PTSD) and complex PTSD (CPTSD). European Journal of Psychotraumatology, 10(1), 1608718. 10.1080/20008198.2019.1608718 31143410 PMC6522970

[bibr18-00207640211057720] Hyland P. Karatzias T. Shevlin M. Cloitre M. (2019). Examining the discriminant validity of complex posttraumatic stress disorder and borderline personality disorder symptoms: Results from a United Kingdom population sample. Journal of Traumatic Stress, 32(6), 855–863. 10.1002/jts.22444 31752053

[bibr19-00207640211057720] Jeffreys M. D. Leibowitz R. Q. Finley E. Arar N. (2010). Trauma disclosure to health care professionals by veterans: Clinical implications. Military Medicine, 175(10), 719–724. 10.7205/MILMED-D-10-00054 20968260

[bibr20-00207640211057720] Karatzias T. Hyland P. Bradley A. Cloitre M. Roberts N. P. Bisson J. I. Shevlin M. (2019). Risk factors and comorbidity of ICD-11 PTSD and complex PTSD: Findings from a trauma-exposed population based sample of adults in the United Kingdom. Depression and Anxiety, 36(9), 887–894. 10.1002/da.22934 31268218

[bibr21-00207640211057720] Karatzias T. Shevlin M. Fyvie C. Hyland P. Efthymiadou E. Wilson D. Roberts N. Bisson J. I. Brewin C. R. Cloitre M. (2017). Evidence of distinct profiles of posttraumatic stress disorder (PTSD) and complex posttraumatic stress disorder (CPTSD) based on the new ICD-11 trauma questionnaire (ICD-TQ). Journal of Affective Disorders, 207, 181–187. 10.1016/j.jad.2016.09.032 27723542

[bibr22-00207640211057720] Kazlauskas E. (2017). Challenges for providing health care in traumatized populations: Barriers for PTSD treatments and the need for new developments. Global Health Action, 10(1), 1322399. 10.1080/16549716.2017.1322399 28562198 PMC5496089

[bibr23-00207640211057720] Kazlauskas E. Gegieckaite G. Hyland P. Zelviene P. Cloitre M. (2018). The structure of ICD-11 PTSD and complex PTSD in Lithuanian mental health services. European Journal of Psychotraumatology, 9(1), 1414559. 10.1080/20008198.2017.1414559 33680347 PMC7874935

[bibr24-00207640211057720] Kazlauskas E. Zelviene P. (2016). Trauma research in the Baltic countries: From political oppression to recovery. European Journal of Psychotraumatology, 7, 29295. 10.3402/ejpt.v7.29295 26996532 PMC4800283

[bibr25-00207640211057720] Kazlauskas E. Zelviene P. Daniunaite I. Hyland P. Kvedaraite M. Shevlin M. Cloitre M. (2020). The structure of ICD-11 PTSD and complex PTSD in adolescents exposed to potentially traumatic experiences. Journal of Affective Disorders, 265, 169–174. 10.1016/j.jad.2020.01.061 32090738

[bibr26-00207640211057720] Kline R. B. (2011). Principles and practice of structural equation modeling (3rd ed.). The Guilford Press.

[bibr27-00207640211057720] Knefel M. Lueger-Schuster B. Karatzias T. Shevlin M. Hyland P. (2019). From child maltreatment to ICD-11 complex post-traumatic stress symptoms: The role of emotion regulation and re-victimisation. Journal of Clinical Psychology, 75(3), 392–403. 10.1002/jclp.22655 29931669 PMC6686279

[bibr28-00207640211057720] Krammer S. Kleim B. Simmen-Janevska K. Maercker A. (2016). Childhood trauma and complex posttraumatic stress disorder symptoms in older adults: A study of direct effects and social-interpersonal factors as potential mediators. Journal of Trauma & Dissociation, 17(5), 593–607. 10.1080/15299732.2014.991861 26011396

[bibr29-00207640211057720] Kvedaraite M. Zelviene P. Elklit A. Kazlauskas E. (2020). The role of traumatic experiences and posttraumatic stress on social anxiety in a youth sample in Lithuania. Psychiatric Quarterly, 91(1), 103–112. 10.1007/s11126-019-09684-7 31773470

[bibr30-00207640211057720] MacGinley M. Breckenridge J. Mowll J. (2019). A scoping review of adult survivors’ experiences of shame following sexual abuse in childhood. Health & Social Care in the Community, 27(5), 1135–1146. 10.1111/hsc.12771 31157486

[bibr31-00207640211057720] Maercker A. Hecker T. Augsburger M. Kliem S. (2018). ICD-11 prevalence rates of posttraumatic stress disorder and complex posttraumatic stress disorder in a German nationwide sample. The Journal of Nervous and Mental Disease, 206(4), 270–276. 10.1097/nmd.0000000000000790 29377849

[bibr32-00207640211057720] Maercker A. Horn A. B. (2013). A socio-interpersonal perspective on PTSD: The case for environments and interpersonal processes. Clinical Psychology & Psychotherapy, 20(6), 465–481. 10.1002/cpp.1805 22730216

[bibr33-00207640211057720] McDonald R. P. (1978). Generalizability in factorable domains: “Domain validity and generalizability”. Educational and Psychological Measurement, 38(1), 75–79. 10.1177/001316447803800111

[bibr34-00207640211057720] Mordeno I. G. Nalipay M. J. N. Mordeno E. R. (2019). The factor structure of complex PTSD in combat-exposed Filipino soldiers. Psychiatry Research, 278, 65–69. 10.1016/j.psychres.2019.05.035 31153009

[bibr35-00207640211057720] Müller J. Maercker A. (2006). Disclosure und wahrgenommene gesellschaftliche wertschätzung als opfer als prädiktoren von PTB bei kriminalitätsopfern. Zeitschrift Fur Klinische Psychologie Und Psychotherapie, 35(1), 49–58. 10.1026/1616-3443.35.1.49

[bibr36-00207640211057720] Owczarek M. Ben-Ezra M. Karatzias T. Hyland P. Vallieres F. Shevlin M. (2020). Testing the factor structure of the international trauma questionnaire (ITQ) in African community samples from Kenya, Ghana, and Nigeria. Journal of Loss and Trauma, 25(4), 348–363. 10.1080/15325024.2019.1689718

[bibr37-00207640211057720] Ozer E. J. Best S. R. Lipsey T. L. Weiss D. S. (2003). Predictors of posttraumatic stress disorder and symptoms in adults: A meta-analysis. Psychological Trauma Theory Research Practice and Policy, 129(1), 52–73. 10.1037/1942-9681.s.1.3 12555794

[bibr38-00207640211057720] Palic S. Zerach G. Shevlin M. Zeligman Z. Elklit A. Solomon Z. (2016). Evidence of complex posttraumatic stress disorder (CPTSD) across populations with prolonged trauma of varying interpersonal intensity and ages of exposure. Psychiatry Research, 246, 692–699. 10.1016/j.psychres.2016.10.062 27839826

[bibr39-00207640211057720] Pielmaier L. Maercker A. (2011). Psychological adaptation to life-threatening injury in dyads: The role of dysfunctional disclosure of trauma. European Journal of Psychotraumatology, 2(1), 8749. 10.3402/ejpt.v2i0.8749 PMC3402151 22893822

[bibr40-00207640211057720] Pineles S. L. Arditte Hall K. A. Rasmusson A. M. (2017). Gender and PTSD: Different pathways to a similar phenotype. Current Opinion in Psychology, 14, 44–48. 10.1016/j.copsyc.2016.11.002 28813318

[bibr41-00207640211057720] Redican E. Nolan E. Hyland P. Cloitre M. McBride O. Karatzias T. Murphy J. Shevlin M. (2021). A systematic literature review of factor analytic and mixture models of ICD-11 PTSD and CPTSD using the international trauma questionnaire. Journal of Anxiety Disorders, 79, 102381. 10.1016/j.janxdis.2021.102381 33714868

[bibr42-00207640211057720] Simon N. Roberts N. P. Lewis C. E. van Gelderen M. J. Bisson J. I. (2019). Associations between perceived social support, posttraumatic stress disorder (PTSD) and complex PTSD (CPTSD): Implications for treatment. European Journal of Psychotraumatology, 10(1), 1573129. 10.1080/20008198.2019.1573129 30788064 PMC6374963

[bibr43-00207640211057720] Truskauskaite-Kuneviciene I. Brailovskaia J. Kamite Y. Petrauskaite G. Margraf J. Kazlauskas E. (2020). Does trauma shape identity? Exploring the links between lifetime trauma exposure and identity status in emerging adulthood. Frontiers in Psychology, 11, 570644. 10.3389/fpsyg.2020.570644 33041939 PMC7522346

[bibr44-00207640211057720] Ullman S. E. Filipas H. H. (2001). Predictors of PTSD symptom severity and social reactions in sexual assault victims. Journal of Traumatic Stress, 14(2), 369–389. 10.1023/A:1011125220522 11469163 PMC3583013

[bibr45-00207640211057720] Weathers F. W. Blake D. D. Schnurr P. P. Kaloupek D. G. Marx B. P. Keane T. M. (2013). The life events checklist for DSM-5 (LEC-5). Instrument available from the National Center for PTSD at www.ptsd.va.gov

[bibr46-00207640211057720] World Health Organization. (2018). International statistical classification of diseases and related health problems (11th ed.). World Health Organization. https://icd.who.int/10.2471/BLT.25.294650 PMC13531092 42683092

[bibr47-00207640211057720] Zelviene P. Kazlauskas E. Maercker A. (2020). Risk factors of ICD-11 adjustment disorder in the Lithuanian general population exposed to life stressors. European Journal of Psychotraumatology, 11(1), 1708617. 10.1080/20008198.2019.1708617 32002141 PMC6968697

